# *In vitro* antitumor capacity of extracts obtained from the plants *Plukenetia volubilis *(Sacha inchi) and *Moringa oleifera* in gastric cancer

**DOI:** 10.12688/f1000research.158563.2

**Published:** 2025-05-27

**Authors:** Jose Vargas, Natalia Arbelaez, Denny Cardenas, Javier Murillo, Victoria Ospina, Sara Robledo, Javier Soto

**Affiliations:** 1 BIOGEN-Universidad de Santander, Facultad de Ciencias Médicas y de la Salud, Instituto de Investigación Masira, Cúcuta, Colombia; 2 PECET-Facultad de Medicina, Universidad de Antioquia, Medellín, Colombia; 3 Grupo Estudios Preclínicos, Corporación CIDEPRO, Medellín, Colombia

**Keywords:** antitumor, apoptosis, cytotoxicity, leaf extract, oil seed, plants

## Abstract

**Background:**

Gastric cancer is the fifth most common cancer and the third leading cause of cancer deaths worldwide. Perioperative or adjuvant chemotherapy improves survival in patients with stage 1B or higher cancers. *Moringa oleifera* and *Plukenetia volubilis* (Sacha inchi) have been reported to enhance various biological functions, including antitumor and antiproliferative activity.

**Methods:**

In order to evaluate this potential present in crude extracts of the leaves of these plants, as well as the seed oil of *P.volubilis*, the antitumor activity was determined according to the effect of these derivatives on different biological parameters such as cytotoxicity, proliferation, cell cycle, apoptosis (among others), in AGS cells (CRL-1739).

**Results:**

All extracts tested were cytotoxic at 90 and 160 μg/ml concentrations *. P. volubilis* seed oil showed 95% mortality at 1% concentration (CC _50_ = 46.7%). Cell proliferation was inhibited, and all extracts affected the cell cycle, but the *P. volubilis* oil significantly induced an accumulation of AGS cells in the sub G1 phase, inducing DNA fragmentation as a mechanism of cell death. The ethanolic *M. oleifera* leaf extract also inhibited cell migration.

**Conclusion:**

*M. oleifera*, *P. volubilis* leaf extracts and *P. volubilis* seed oil can potentially be antitumor products. Further validation in a murine model of gastric cancer is needed to investigate the antitumor potential of these extracts further and to continue the development of herbal products that can help in the management of this type of tumor.

## Introduction

More than 19 million people had their first case of cancer, and nearly half of them died in 2022 ( WHO, 2024). Gastric or stomach cancer is the fifth most common cancer worldwide, with more than one million cases annually, of which 70% of patients die ( Ferlay et al., 2021), mainly because diagnoses occur at late stages and because of this, the probability of success of conventional therapies at these stages is low, making it the fourth most lethal neoplasm globally ( Sung et al., 2021). Despite the efforts made in the last four decades, the cost-benefit and long-term survival picture for many cancer pathologies remains bleak. Between 1971 and 2007, an increase in survival of only 17% has been achieved in ovarian cancer ( Lloyd, Cree, & Savage, 2015), while in breast cancer, the increase has been 38% in 10-year survival. Significant barriers to major advances include low rates of early detection, lack of effective prognostic and predictive strategies, and the emergence of chemoresistance, which ultimately leads to patient death.

Canonical chemotherapy for the treatment of gastric cancer is primarily based on combinations of cisplatin and 5-fluorouracil (5-FU) or its derivatives, such as oxaliplatin and capecitabine. The genotoxic effects of chemotherapy and radiotherapy are the same as those that lead to the initiation and maintenance of cancer, and it is puzzling that genotoxic agents are given preference in cancer treatment over other substances that may act more specifically. Approximately 25% of all new anticancer drugs approved in the last 30 years are related to natural products ( Newman & Cragg, 2020). In addition, such natural compounds obtained through diet offer options for preventing and treating many diseases, including cancer ( Cragg & Pezzuto, 2016). Natural products have been so successful that they have doubled human life expectancy in the 20th century. For more than five decades, they have been positioned as weapons in the battle against cancer, thanks to the presence of exotic structures rich in functional groups ( Verdine, 1996). About 1 million natural products, of which more than half come from plants, are the most critical anticancer products ( Demain & Vaishnav, 2011).

Given the characteristics of natural products, many studies have focused on uncovering their therapeutic potential in cancer research. An example of this is the study of extracts from the *Moringa oleifera*, known as ‘the tree of life,’ a tropical and subtropical plant with several recognized biological properties, mainly anti-inflammatory ( Cheenpracha et al., 2010). Regarding its antitumor capacity, several studies have been carried out based mainly on extracts from the leaf in ovarian, prostate, and breast cancer tumor lines ( Al-Asmari et al., 2015; Del Mar Zayas-Viera, Vivas-Mejia, & Reyes, 2016; Ghosh, 2013), hepatocarcinoma and leukemia ( Khalafalla et al., 2010), multiple myeloma ( Parvathy & Umamaheshwari, 2007), KB human tumor lines ( Sreelatha, Jeyachitra, & Padma, 2011) and those derived from esophageal cancer ( Tiloke, Phulukdaree, & Chuturgoon, 2016), colorectal cancer ( Al-Asmari et al., 2015), as well as in animal approaches using the Ehrlich solid tumor model ( W. K. Khalil, Ghaly, Diab, & ELmakawy, 2014), among other studies ( Khor, Lim, Moses, & Abdul Samad, 2018).

*Plukenetia Volubilis* is another plant on which the study of its components in cancer has focused, although not as extensively as *Moringa.* *P. volubilis* is commonly known as Sancha inchi (SI). It is distributed along the western and northern edge of the Amazon basin, through Brazil, Bolivia, Peru, Ecuador, Colombia, Venezuela, and Suriname, and in the Lesser Antilles ( del-Castillo, Gonzalez-Aspajo, de Fátima Sánchez-Márquez, & Kodahl, 2019). In recent years, *P. volubilis* has attracted attention because of the abundance and composition of its seed oil, which is now commercially available. Although the biological function of SI has not been fully delineated, its beneficial impact in modulating non-communicable diseases has gained popularity worldwide for its antioxidant, anti-inflammatory, and immunomodulatory properties, mainly from leaves and fruit hulls ( Nascimento et al., 2013; Wuttisin, Nararatwanchai, & Sarikaphuti, 2021). It is also recognized because its consumption has been associated with the prevention of cardiovascular diseases, inflammatory diseases, dermatitis, and control of tumor proliferation, especially given its recognized high content of essential fatty acids, as well as the hypolipidemic ( Cárdenas, Gómez Rave, & Soto, 2021) and antitumor activity in cervical and lung tumor lines ( Nascimento et al., 2013). It is also recognized as a sustainable crop ( Kodahl & Sørensen, 2021).

There is a growing interest in different areas about the potential of promising plants, including *Plukenetia volubilis* and *Moringa oleifera*, but there remains a lack of focused studies evaluating their specific effects on gastric cancer, especially for *P.volubilis*, and particularly with regard to its unprocessed seed oil. For this plant there is research predominantly focused on its nutritional value and general antioxidant or anti-inflammatory properties, with only limited exploration into its potential to modulate cancer-related pathways such as apoptosis, cell cycle arrest or proliferation inhibition in gastric tumors, in fact, the direct impact of its components in this context is still unknown. This represents a critical gap, particularly given the increasing global burden of gastric cancer and the need for novel, plant-derived chemopreventive agents.

The selection of *M. oleifera* and *P. volubilis* for this study was based on their traditional medicinal use, promising bioactivity, and particularly their diverse phytochemical profiles, which support potential anticancer properties. *M. oleifera* leaves are rich in flavonoids (e.g., quercetin, kaempferol), glucosinolates, isothiocyanates (e.g., niazimycin, benzyl isothiocyanate), phenolic acids, and alkaloids, compounds that have demonstrated antiproliferative, antioxidant, and pro-apoptotic effects in various tumor models, including breast, colon, prostate, and leukemia cell lines ( Chiș et al., 2023; Pop, Kerezsi, & Ciont, 2022). *P. volubilis*, although less extensively studied, has attracted attention for its seed oil’s high content of polyunsaturated fatty acids (especially α-linolenic and linoleic acid), as well as tocopherols, phytosterols (β-sitosterol, stigmasterol), terpenoids, and phenolic ( Chirinos et al., 2013; Đurović, Radovanović, Tomić, Marjanović, & Mandić, 2025; Valencia, Romero-Orejon, Viñas-Ospino, & Barriga-Rodriguez, 2021).

Regarding *M. oleifera* previous studies have shown that its extracts, especially from leaves and seeds, induce apoptosis, inhibit cell proliferation and generate DNA damage in different tumor cell lines such as breast, colon and lung cancer ( Al-Asmari et al., 2015; Kuete et al., 2011; Sreelatha et al., 2011; Tiloke, Phulukdaree, & Chuturgoon, 2013). Despite these findings, the effect of *M. oleifera* on gastric cancer remains poorly explored, despite the fact that this neoplasm represents one of the leading causes of cancer mortality worldwide. For such reason, it is a priority to investigate the antitumor potential of *Moringa oleifera*-derived compounds in in vitro models of gastric cancer. A preliminary study by Kuete et al. (2011) showed that methanolic extracts of *M. oleifera* exert cytotoxic activity against AGS (human gastric adenocarcinoma) cells, suggesting a possible direct effect on cell viability ( Kuete et al., 2011). However, the molecular mechanisms involved in this cytotoxicity have not yet been thoroughly characterized.

The species tested in this study are adaptable to tropical climates and easy to cultivate, which allows large-scale production in regions of Latin America, Africa and Asia. This facilitates continuous access to plant biomass for extraction and analysis of bioactive compounds, reducing research and development costs. In addition, both plants have a history of traditional use as foods or supplements, suggesting a favorable safety profile. This makes their compounds good candidates for preclinical studies with lower risk of serious adverse effects compared to synthetic agents. The fact of considering promising plants such as these in this type of studies encourages ethnobotanical and biotechnological research in regions with high biodiversity, promoting local scientific development and the sustainable use of endemic natural resources.

Accordingly, this work aimed to study the effect of extracts obtained from these two plants on cytotoxicity, inhibition of cell proliferation and migration through *in vitro* assays in the AGS tumor line, as well as the study of the possible mechanism responsible for these effects, seeking to advance in the development of a phytotherapeutic approach for gastric carcinoma.

## Methods

### Plant material and extracts

Dehydrated leaves (1.5 kg) of *P. volubilis* (Sacha inchi) and *Moringa oleifera* were obtained from hydroponic cultures (Sasha Colombia SAS, Piedecuesta, Colombia). The seeds of *P. volubilis* were also included. All materials were processed at the Chromatography and Mass Spectrometry Laboratory, CROM-MASS- UIS (Bucaramanga, Colombia). Extractions were performed using rotary evaporation with three solvents: petroleum ether (PE), cyclohexane (CH), and ethanol (EtOH). For each solvent, the following were used: PE: 1.5 L, extracted for 24 hours; CH: 1.5 L, extracted for 24 hours and EtOH: 1.5 L, extracted for 48 hours. The resulting products were freeze-dried and stored at 4 °C until use. Dimethyl sulfoxide (DMSO) at 0.2% (Scharlau Química, SU01590250) was used as the solubilization vehicle. The vegetable oil from *P. volubilis* was obtained by cold pressing the seeds, followed by solubilization of the oil in a mixture of 1.25% ethanol and RMPI medium (Sciencell, 09521). Although the crude extracts initially exhibited coloration, the working dilutions used for all spectrophotometric assays were visually colorless or displayed negligible coloration that did not interfere with absorbance readings.

### Cell line and culture

The non-metastatic gastric cancer tumor line (ATCC CRL-1739) was maintained in RPMI (Sciencell, 09521) and supplemented with 10% SFB (BIOWEST, S181B-500) and a cocktail of antibiotics and antifungal (10. 000 units/mL penicillin, 10,000 μg/mL streptomycin and 25 μg/mL amphotericin B) (Sciencell, 0533)) and incubated at 37°C in 5% CO _2_ atmosphere until the sufficient confluence of cells was achieved.

### Cell cytotoxicity

The effect of the extracts of *M. oleifera* and *P. volubilis*, as well as the *P. volubilis* oil were evaluated according to the viability of AGS cells after treatment by 3-(4,5-dimethylthiazol-2-yl)-2,5-diphenyltetrazolium bromide (MTT) assay ( Selvakumaran, Pisarcik, Bao, Yeung, & Hamilton, 2003), which is a colorimetric assay that assesses the cellular metabolic activity of NADPH-dependent mitochondrial oxidoreductase enzymes, which can reduce tetrazolium salts (yellow) to formazan (purple). Briefly, cells were seeded in 96-well plates in quadruplicate at 20,000 cells/well density, reaching the optimal population after 48 hours. The cells were treated with six different concentrations of each plant extract and *P. volubilis* oil for 72 hours. The concentrations tested for the extracts were 6.25, 12.5, 25, 50, 100, and 200 μg/mL, while the oil was tested at concentrations of 1.6, 6.3, 25, and 100% v/v. Once the treatment was completed, the MTT test was performed according to the instructions of the commercial company (MTT Assay Kit Cell Proliferation, ABCAM, ab211091). Briefly, 50 μL of serum-free media and 50 μL of MTT reagent were added to each well and the cells were incubated at 37°C for 3 hours. After incubation the MTT reagent-supplemented media was removed and 150 μL of MTT solvent were added to each well. The absorbance was determined at 590 nm in a multimodal plate reader (Varioskan Flash, Thermo Fisher Scientific, USA). Doxorubicin (Merck, 1225703), cisplatin (Seven Pharma M000629), and miltefosine (Abcam, ab143837) were positive controls for cytotoxicity. The assays were performed in three independent experiments with two replicates per assay. Values were normalized according to the untreated control. Results are presented as the Cytotoxic Concentration 50 (CC50) of the treatments for AGS cells (CC _50_), which was determined by sigmoidal regression using Msxlfit software (GO Business Solution, Guildford, UK). Two experiments were performed, each treatment in triplicate.

The effect of the extracts on AGS cell viability/death is expressed as the percentage of viability using the following formula:

Cytotoxicity(%)=[100−(A590of treated cells/A590of control cells)×100]

### Cell proliferation

The proliferation of AGS cells cultured with the extracts of *P. volubilis* and *M. oleifera* leaves, and *P. volubilis* oil was determined using the CellTiter-Blue ^®^ kit (Promega, G8080). An inoculum of 5,000 cells/mL per well grew for 24 hours to allow cell adhesion. Cells were then exposed to 100, 50, 25, and 10 μg/mL concentrations of extracts and oil, and serial readings were taken every 48 hours until 96 hours post-treatment. Test compounds and controls were added to get a final volume of 100 μL in each well. 20 μL/well of CellTiter-Blue ^®^ Reagent were added after the desired test exposure. After 4 hours of incubation the fluorescence was quantified in a Varioskan™ LUX microplate reader (Thermo Scientific™) at excitation/emission wavelength 460/590 nm, and assays were performed in triplicate.

### Cell cycle evaluation

AGS cells were seeded in 24-well plates at 1×10 ^5^ cells/well density for cell cycle analysis and cultured overnight. Each extract/oil/control was added at the concentration equivalent to the respective CC _50_. After eight hours of incubation at 37 °C, 5% CO _2_, cells were mechanically detached with a syringe plunger, and cells were collected and fixed with 95% ethanol and stored at -20°C overnight ( Hitora et al., 2021). The cells were washed with cold PBS and incubated in 400 μL of a solution containing PI (propidium iodide) (ThermoFisher, BMS500PI) at 50 μg/mL, RNase A (ThermoFisher R1253) (100 μg/mL), EDTA (Ethylenediaminetetraacetic acid) solution (Sigma-Aldrich E8008) (0.5 mM), and Triton X-100 (0.2%) (Sigma-Aldrich, T9284) for 30 min at 37 °C. PI fluorescence of the cell suspension was analyzed for cellular DNA fragmentation on an LSR Fortessa™ cytometer (Becton Dickinson BD Biosciences, USA). Data were obtained using FlowJo 7.6.2 data analysis software (FlowJo, USA). Hypodiploid (sub-G1 phase) cells were used as a marker for DNA fragmentation (apoptotic cells). The sub-G1 phase population was subtracted from the total number of events, and cell cycle analysis was performed by Dean Jett Fox analysis (RMS<10).

### Assessment of oxidative stress

ROS production in AGS cells treated or not with *P. volubilis* seed oil was measured using H2DCFDA (2′,7′dihydrofluorescein) (ThermoFisher, D399) in AGS cells. The experiment was performed in 24-well plates using 2 × 10 ^5^ cells per well; treatments were administered at CC _50_, and PHA (phytohemagglutinin) (Sigma-Aldrich, L8902) at 20 μg/mL was used as a control for ROS production. Kinetics was performed at 12, 24, and 48 h post-treatment; after the incubation time, the medium was removed, and 400 μL of H2DCFDA solution at 5 μM was added, incubating for one hour at 37 °C; cells were then washed and resuspended in 250 μL of PBS. After this, the cells were mechanically detached and transferred to a 96-well plate and finally read by Cytomics FC 85 500MPL flow cytometry, Brea, CA, at 488 nm excitation and 525 nm emission using argon laser and counting 10.000 events ( Zapata et al., 2020).

On the other hand, ON production in AGS cells treated or not with *P. volubilis* seed oil was performed in 24-well plates with a cell density of 2 × 10 ^5^ cells per well; the treatments were subsequently administered in 10% SFB supplemented RPMI-1640 medium at CC _50_; PMA (Phorbol 12-myristate 13-acetate) (Merck, 16561-29-8) at 1 μg/mL was used as a control for ON production, and readings were taken at 24, 48 and 72 h post-treatment. After the end of the incubation period, the medium was removed, and 400 μL of DAF-FM diacetate (4-amino-5-methylamino-2 ′,7′-difluorofluorescein diacetate) probe (ThermoFisher, D23844) was added at 5μM in RPMI-1640 without phenol red, this was incubated for one hour at 37°C. After the time was up, the cells were washed and resuspended in 250 μL of PBS and detached with a syringe plunger. Finally, the contents were transferred to 96-well plates and read by Cytomics FC 500MPL flow cytometry, Brea, CA, at 488 nm excitation and 525 nm emission using an argon laser and counting 10,000 events. The number of positive cells was determined ( Zapata et al., 2020).

### Caspase activity

The activity of caspases as apoptotic markers was determined using the commercial Caspase 3, Caspase 8, and Caspase 9 Multiplex Activity Assay Kit (Abcam, ab219915). Briefly, 20,000 cells were seeded in 96-well plates for 24 hours until adhesion was achieved. Cells were treated with the CC _50_ extracts and oil and incubated at 37 °C, 5% CO _2_, and 95% of humidity. The cells were treated for 24, 48 and 72 hours and at the end of the treatment time, 100 uL of previously prepared Caspase assay loading solution was added to each well directly to the cell plate without removing culture media/treatment. Subsequently, the plates were incubated for one hour at room temperature protected from light and after this time the caspases activity was monitored. Fluorescence was measured at excitation/emission wavelengths of 535/620 nm (Caspase 3), 490/525 nm (Caspase 8), and 370/450 nm (Caspase 9).

### Annexin V activity

Death mechanisms were analyzed through the performance of the commercial Real-Time Apoptosis and Necrosis Assay kit (Promega, JA1011). AGS cells (10,000 cells/mL) were seeded in 96-well plates for 24 hours until adherence. Cells were treated for 24, 48 and 72 hours with CC _50_ of both extracts and oil and at the end of the treatment time, 100 ul of previously prepared 2X Detection Reagent were added to each well. Subsequent incubation was carried out in a Varioskan™ LUX microplate reader (Thermo Scientific, USA) at 37 °C, 5% CO _2_, and 95% humidity for three days, with readings taken every 24 hours. Phosphatidylserine translocation was measured by detecting a luminescence signal (Beads/s, integration time 1000 ms) produced by annexin V-dependent assembly of two luciferase fragments. Membrane integrity was measured as fluorescence at excitation/emission wavelengths of 485/525 nm. DMSO 25% was used as a control for apoptotic induction.

### Wound recovery assay

To examine whether treatment affects cell proliferation and migration, 250,000 cells/well were seeded in 24-well plates in 500 μl of growing medium. Once 80% confluency was reached, the monolayer was deliberately wounded vertically with a micropipette tip, the culture medium was changed, and the cells were seeded with the respective extract and oil at the respective CC _50_. Microphotographs were taken with a magnification of 40× at 0h, 24h, and 48h to assess wound closure. A straight line was drawn with an ultra-fine tip marker on the back of the wells to keep the field during image acquisition. The microscope used was the DMi1 (Leica Microsystems, Germany). The negative control corresponded to cells seeded in 10% FBS RPMI medium without any treatment.

### Statistical analysis

Data are presented as mean value ± standard deviation. Cytotoxicity values are expressed as mean Cytotoxic Concentration (CC _50_) calculated by linear regression analysis with GraphPad Prisma 8.0. The normality test was performed with the Shapiro-Wilk test. Differences in cell cycle were performed with the non-parametric Mann-Whitney U test.) Statistically significant differences were established with a p-value <0.05. The effect of treatment on proliferation, cell death, and caspase expression in AGS cells was assessed by comparing the relative fluorescence units by one-factor analysis of variance (ANOVA) after checking the assumptions of normality and homogeneity of variances. Where normality was not accepted, the non-parametric Kruskal-Wallis test was used. Analyses were performed in IBM SPSS Statistics software, version 25.0, using a 95% confidence level in all cases. Where significant differences were identified, post hoc tests were performed using the Tukey or Games-Howell test, as appropriate *.*

## Results

### Cytotoxic effect of *Plukenetia volubilis* and *Moringa oleifera* derivative extracts in AGS tumor cells

**Figure 1.**
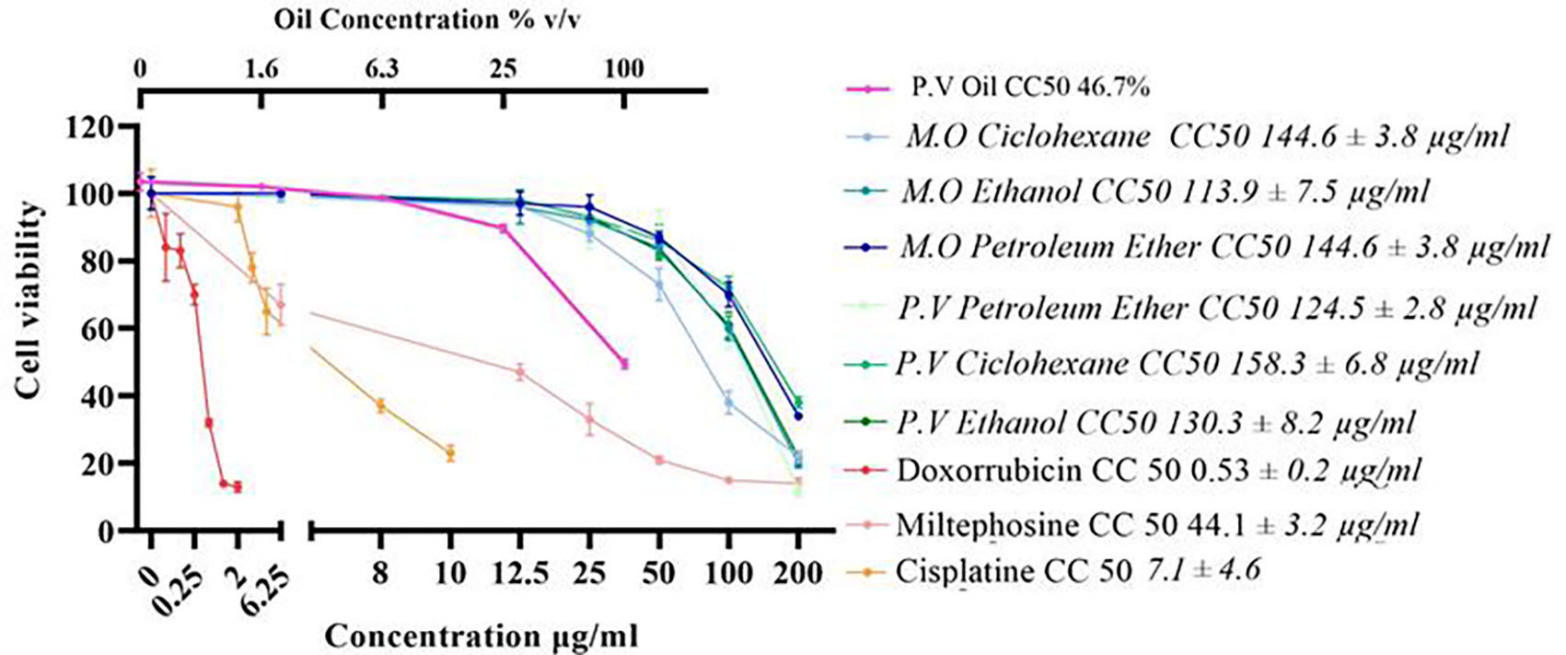
Effect of derivatives obtained from each of the plants under study on cell viability. Data were normalized with respect to the untreated control (100% survival) and are shown as the mean and standard deviation of at least three independent experiments performed in quadruplicate for each dose. The cytotoxic concentration 50 (CC _50_) of each derivative is shown.

Cells were exposed to various concentrations of the extracts and oil for 72 hours. The effect on viability was evidenced by the MTT assay. The results revealed that the extracts of both plants affect the survival of the AGS gastric cancer cell line, with CC _50_ values ranging from 94.5 mg/mL to 158.3 μg/mL ( Figure 1). The extracts showed a CC _50_ above 100 μg/mL for AGS cells, except for the *M. oleifera* cyclohexane extract, which showed a CC _50_ of 94.3 μg/mL. Given the CC _50_ values obtained for the extracts on AGS cells, it can be deduced that, except for the cyclohexane extract of *M. oleifera*, which showed high cytotoxicity, the extracts present moderate toxicity to these cells ( Figure 1). Similarly, the oil obtained from *P. volubilis* seed also showed cytotoxic potential on AGS cells with a reduction in cell viability of 47% ( Figure 1).

The CH extract from *M. oleifera* was 1.6 times more effective than its counterpart from *P. volubilis*, while the extract obtained with PE was the most cytotoxic. However, at the highest dose tested, the latter induced more cell death (88%) than CH extract (72%) despite having the lowest CC _50_. It was also observed that the cytotoxic effect of the extracts and the oil is dose-dependent, as detailed in the viability curves ( Figure 1).

### Antiproliferative effect of *Plukenetia volubilis* and *Moringa oleifera* derivative extracts in AGS tumor cells

The effect on cell proliferation was determined by cell viability assay with the CellTiter-Blue ^®^ kit (Promega). All derivatives, extracts, and oil affected the proliferation of tumor cells at concentrations below the CC _50_, and this effect is maintained over time.

**Figure 2.**
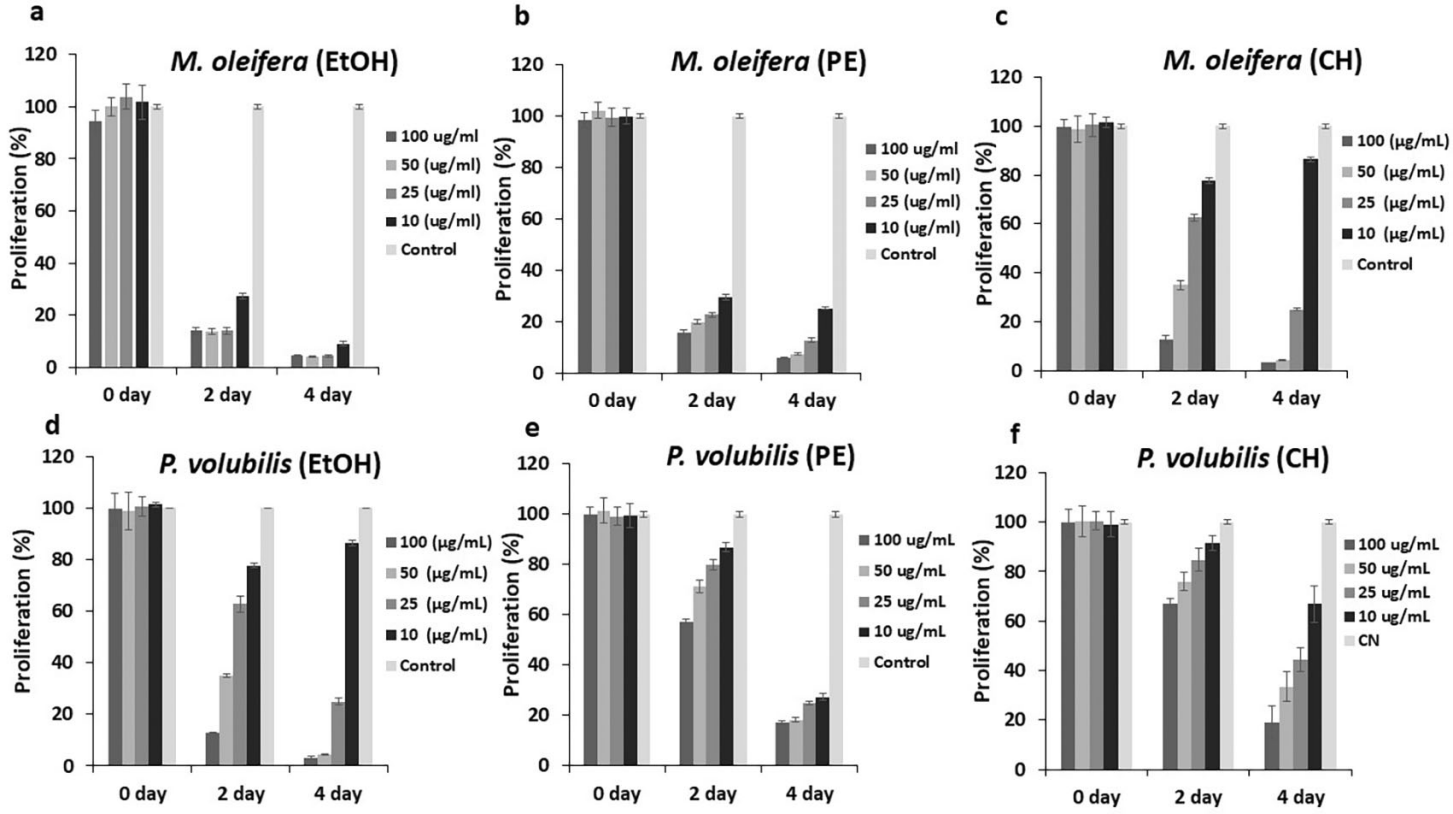
Effect of *M. oleifera* and *P. volubilis* extracts on cell proliferation. Data were normalized with respect to the untreated control (100% survival) and are shown as the mean and standard deviation of at least two independent experiments performed in triplicate for each dose.

*Moringa* extracts had better inhibitory dynamics for the doses required and the time employed compared with *P.volubilis* extracts. For example, the EtOH and PE extracts of *M. oleifera* exhibited a significant inhibitory effect from the second day of treatment, compared to the same extracts from *P. volubilis* ( Figure 2a, b, d, e). Moreover, among all extracts tested, the EtOH extract of *M. oleifera* showed almost complete inhibition at the evaluated concentrations ( Figure 2a). In contrast, the antiproliferative effect of the cyclohexane of *M. oleifera* is not as evident since it was found that the lowest dose of this extract showed an innocuous effect on the AGS line ( Figure 2c). This behavior was also observed in the EtOH extract from *P.volubilis* where the 10 ug/mL dose also had no effect on inhibition of cell proliferation ( Figure 2d). Despite this, it is this extract that exhibits the most significant antiproliferative potential among the *P. volubilis* group, first, because it achieves an evident antiproliferative effect up to a dose of 50 ug/mL, and second, the inhibition was observed from the second day of treatment, events that do not occur with the other two extracts ( Figure 2d, e, f).

**Figure 3.**
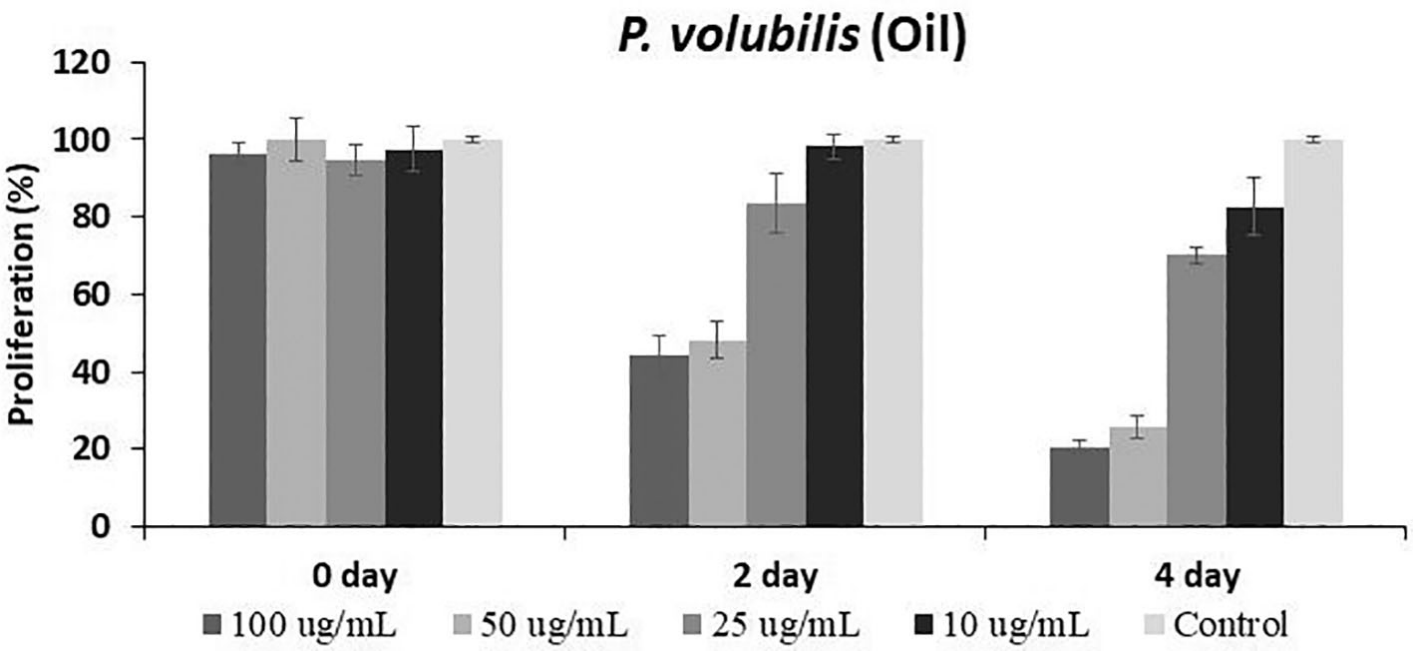
Effect of *P. volubilis* oil on cell proliferation of the AGS line. Data were normalized with respect to the untreated control (100% survival) and are shown as the mean and standard deviation of at least two independent experiments performed in triplicate for each dose.

On the other hand, the *P. volubilis* oil generated a varied inhibitory effect on proliferation at all concentrations tested, especially at the highest concentration ( Figure 3); moreover, the effect was maintained until the end of the treatment.

### Death inducing effect of *Plukenetia volubilis* oil in AGS tumor cells

**Figure 4.**
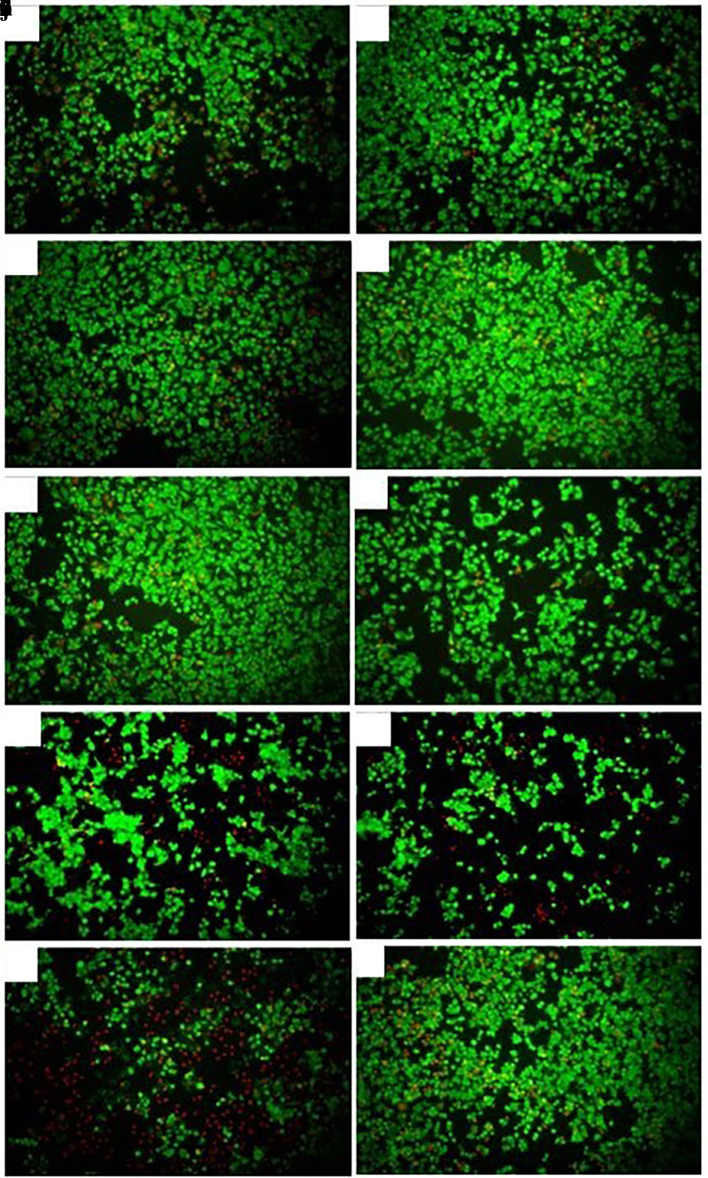
Effect of extracts and oil on the viability/cytotoxicity of AGS cells. The figure shows a representative photograph of AGS cells treated with cyclohexane (a), ethanol (b), and petroleum ether (c) extracts of *P. volubilis.* Photographs (d), (e) and (f) correspond to AGS cells treated with cyclohexane, ethyl and petroleum ether extract of *M. oleifera*, respectively. Cells treated with *P. volubilis* oil 25% v/v (g), N-ethyl Nitrosurea 25% v/v (h), Doxorubicin 5μg/mL (I) and untreated (J): fluorescence microscopy, magnification 10×.

Fluorescence microscopy revealed that cells treated with the different derivatives and N-ethyl nitrosourea showed a generally lower proportion of dead cells (red fluorescence) relative to live cells (green fluorescence), as shown in Figure 4. The viability of AGS cells treated with the extracts and oil at the respective CC _50_ ranged from 43% to 69%, with the CH and EtOH extracts of *M. oleifera* affecting cell viability the most, with viability percentages close to 40%. All extracts produced a low mortality of AGS cells, with the percentages obtained being less than 10% ( Table 1). On the other hand, *P. volubilis* oil at a concentration of 25% showed viability percentages of 77% and mortality of 23%, being the highest mortality of all the plant derivatives evaluated ( Table 1, Figure 4). N-ethyl nitrosurea, used to induce stomach cancer in C57Bl6 mice, produced 89% viability and 9% mortality. With doxorubicin, viability was observed in 38% of cells, and mortality was 66%. These results suggest that *P. volubilis* oil is cytotoxic to AGS cells at concentrations of 25% v/v or higher. As expected, most cells of the untreated group show fluoresce green (live), and only a small proportion of cells show red fluoresce ( Figure 4j).

**Table 1.** Effect of *Plukenetia volubilis* and *Moringa oleifera* extracts on the viability of AGS cells.

Treatment	% live cells	% dead cells
*P. volubilis* CH	68.5 ± 6.8	9.7 ± 1.2
*P. volubilis* EtOH	59.0 ± 2.5	8.6 ± 1.5
*P. volubilis* PE	58.9 ± 1.9	8.4 ± 2.3
*M. olífera* CH	43.5 ± 3.3	1.6 ± 0.3
*M. olífera* EtOH	47.7 ± 8.8	4.7 ± 1.8
*M. olífera* PE	69.7 ± 4.0	7.6 ± 2.4
*P. volubilis* oil (25% v/v)	77.1 ± 1.2	22.6 ± 0.5
N-ethyl nitrosurea (25% v/v)	88.8 ± 0.9	8.6 ± 0.6
Doxorrubicin (5 μg/mL)	37.8 ± 3.7	65.8 ± 8.2
DMSO 20%	52.5 ± 0.22	39.3 ± 10.6
Nontreatment	99.5	0.5

Data represent the mean values + standard deviation of the viability and mortality percentages obtained for each treatment in two trials with three replicates.

### Effect of *P. volubilis* oil in the AGS cell cycle

**Figure 5.**
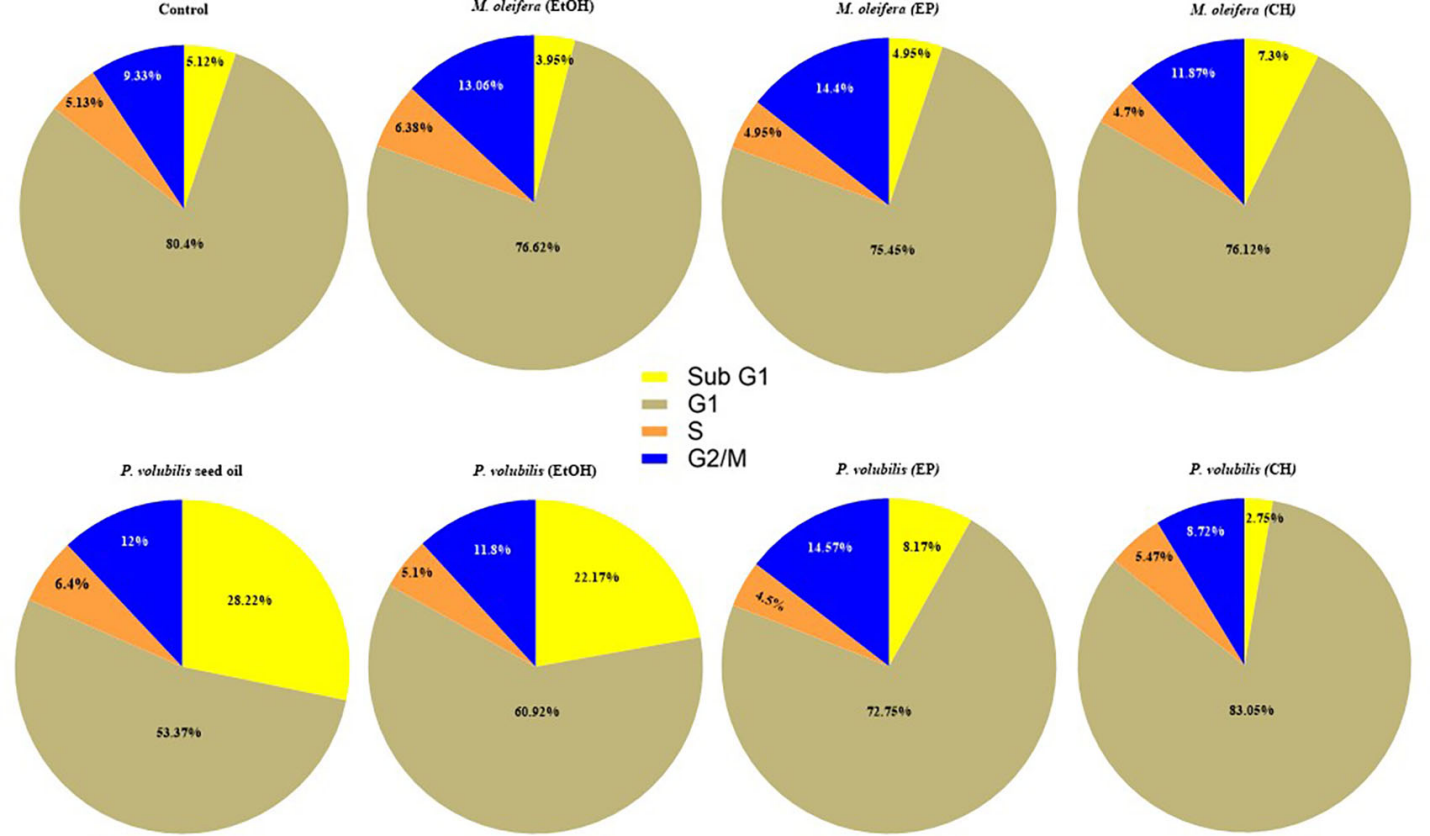
Effect of extacts and oil on the cell cycle of AGS cells. Data correspond to the X ± SD of the percentages of AGS cells treated for 8 hours with *P. volubilis* and *M. oleifera* leaf extracts and *P. volubilis* oil at the corresponding CC _50_. Statistical test ANOVA two-way comparison multiple treatments, Dunnet statistical test p<0.0001 data analyzed in Graph Pad Prisma 80. A value of p>0.05 (NS), p<0.05 (*), p<0.001 (**), and p<0.001 (***) was considered statistically significant (ANOVA) to compare treatments after confirming the normal distribution of the data. Sub G1 cells: hypo diploid cell population (apoptosis); G1 cells: diploid cell population or in G0 - G1 phase; G2/M cells: tetraploid cell population; and S cells: cell population in synthesis phase.

The effect on the content of the DNA in AGS cells treated with the different extracts of *M. oleifera* and *P. volubilis* and the oil of *P. volubilis* was analyzed by flow cytometry with PI staining. The assays revealed statistically significant changes only in cells treated with *P. volubilis* oil compared to untreated cells. In cells treated with *P. volubilis* oil, the percentage of cells in the sub-G1 phase increased from 5.1 ± 2.2% in control cells to 28.2% ± 12.3 within 8 hours of treatment (p = 0.0027), and the percentage of cells in G1 phase decreased from 80% to 53% ± 12.8% (p = 0.0025). With leaf extracts, no statistically significant differences in arrest were observed in the sub-G1 phase cell population compared to untreated cells (p > 0.005) ( Figure 5).

### Effect of *M. oleifera* petroleum ether and *P. volubilis* ethanolic extracts in the AGS cell membrane.

**Figure 6.**
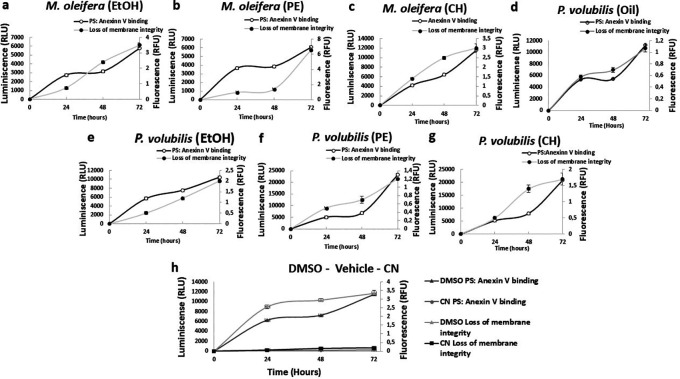
Assessment of apoptosis/necrosis induced by plants derivatives on AGS cells. The figure shows the mean and standard deviation of luminescence and fluorescence of at least two independent experiments performed in sextuplicate for the corresponding CC _50_ for each extract or oil compared to untreated cells as negative control (NC). DMSO was used as a positive control of the assay (h). In panels b and e, there is a significant delay between the appearance of PS: Annexin V binding and the loss of membrane integrity, suggesting an apoptotic phenotype leading to secondary necrosis.

AGS cell death after treatment with *M. oleifera* and *P. volubilis* derivatives extracts was characterized by measuring phosphatidylserine translocation and cell membrane permeability, which occur sequentially during apoptosis. The PE extract of *M. oleifera* and the EtOH extract from *P. volubilis* induced phosphatidylserine externalization over time, with membrane permeability increasing between 48 and 72 hours of treatment ( Figure 6b, e). In contrast, the remaining extracts showed a reversal in the maintained curves over time, increasing progressively. The *P. volubilis* oil induced a membrane permeability greater than the phosphatidylserine translocation ( Figure 6d).

### Effect of *M. oleifera* and *P. volubilis derivatives* in the activation of the caspases in AGS cells

**Figure 7.**
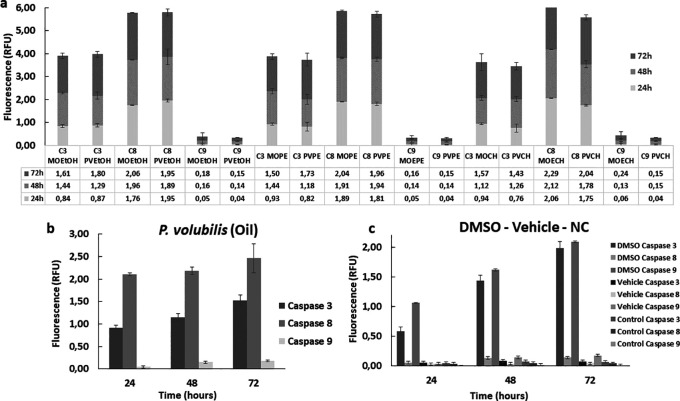
Enzymatic activity of caspases 3, 8, and 9 in tumor cells treated withplants derivatives. The figure shows the mean and standard deviation of the fluorescence of at least two independent experiments performed in sextuplicate for the corresponding CC _50_ of each plant derivative, a) extract or b) oil. Panel c shows the caspase activity on positive (DMSO) and negative (NC, Vehicle) controls. A higher activity is observed in the group treated with the apoptosis inducer regarding the three caspases in a gradual manner over time compared to the negative control and the vehicle. MOEtOH: *M.oleifera* ethanol extract; PVEtOH; *P. volubilis* etanol extract; MOPE: *M.oleifera* petroleum ether extract; PVPE; *P. volubilis* petroleum ether extract; MOCH: *M.oleifera* ciclohexane extract; PVCH; *P. volubilis* ciclohexane extract; NC: negative control (untreated cells).

To complement the study of the effector mechanisms of cell death, the enzymatic activity of some apoptotic markers, such as caspases 3, 8, and 9, was evaluated. Overall, all extracts increased the enzymatic activity of the caspases studied in treated AGS cells compared to the untreated control. This activity increased gradually in all treated groups and remained consistent for 72 hours. Both extracts and oil induced higher caspase eight activity compared to caspases 3 and 9 ( Figure 7a). The CH extract from *M. oleifera* and *P. volubilis* showed the most significant effect on caspase 8 induction, but it was the oil the derivative that induced the highest caspase 8 activity in AGS cells at 72 hours ( Figure 7b). On the contrary, caspase 9 showed the lowest activity in the different treatments, exhibiting almost identical fluorescence in all derivatives, except for the *M. oleifera* CH extract ( Figure 7a), which induced a discrete increase in the expression of caspase 9 compared to the other treatments.

### Effect of treatment of *Plukenetia volubilis* oil in the production of ROS and NO by AGS tumor cells

The *P. volubilis* oil increased the production of ROS and NO in AGS cells compared to untreated cells ( Table 2). The differences between untreated and extract-treated cells were statistically significant (p = 0.037). No statistically significant differences were observed between the stimulation of cells with PHA (used as a positive control for ROS production) or PMA (used as a positive control for ON production) and treatment with *P. volubilis* seed oil.

**Table 2.** Effect of *Plukenetia volubilis* seed oil on ROS and ON expression by AGS cells.

Treatment	ROS	ON
C-	29.3 ± 2,6	22.3 ± 1.7
C+	63.1 ± 13.6 (↑ 2.1 x)	58.3 ± 6.5 (↑ 2.6 x)
*P. volubilis* oil	46.7 ± 10.8 (↑ 1.6 x)	50.1 ± 2.7 (↑ 2.2 x)

Data show the mean ± SD of the percentage of cells positive for ROS and ON without treatment (C-), treated with PHA (for ROS or PMA (for ON) (C+), and treated with *P. volubilis* seed oil. Arrows ↑↓ represent the increase or decrease concerning the untreated basal condition.

### Inhibition of cell migration

**Figure 8.**
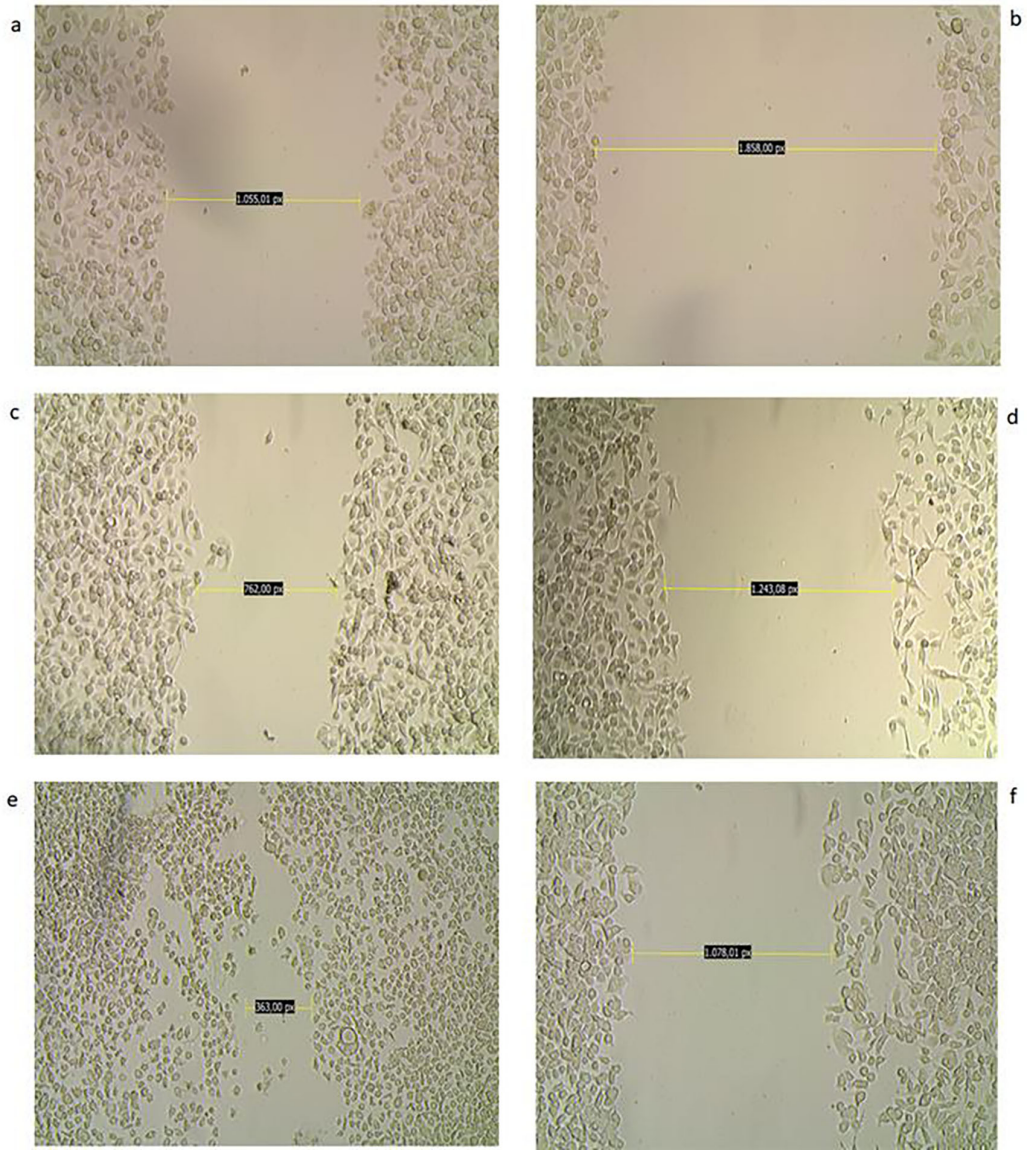
Inhibition of migration of AGS cells. The figure shows a photomicrograph of the scraping at 0 (a,b), 24 (c,d), and 48 (e,f ) hours: a,b: Wound in the monolayer of AGS cells at 0 hours c: Wound area in control (untreated) cells shows no significant wound closure at 24 hours. e: At 48 hours, the wound has almost healed in control cells. d,f: Wounds in treated cells (CC _50_ dose) healed at a slower rate, with an almost negligible decrease in the area studied; wounds in treated cells remained with few cells within the area studied at 24 and 48 hours, magnification 10×.

The potential of biological derivatives to inhibit the migration ability of AGS cells was studied by analyzing the closure between the edges of the deliberately provoked wound in the cell monolayer. This analysis was performed over time and evaluated qualitatively. Of all the derivatives studied, the ethanolic extract at a concentration of 100 μg/mL revealed through microphotographs a difference with respect to its untreated counterpart in terms of wound closure at the end of treatment after 48 hours. Comparative analysis of the two groups over time allows us to identify a clear difference in the progress of wound closure in the untreated cells, which is synonymous of the maintenance of their migratory capacity, unlike what was observed in the treated tumor cells ( Figure 8).

## Discussion

The present study provides new insights into the in vitro antitumor potential of *Moringa oleifera* and *Plukenetia volubilis* extracts against gastric cancer cells. Our results showed significant cytotoxic and antiproliferative effects across all tested derivatives, supporting the role of these plant species as promising sources of bioactive compounds with therapeutic potential. Notably, the unprocessed seed oil of *P. volubilis* exhibited a pronounced cytotoxic effect, inducing DNA fragmentation and apoptosis, and representing, to our knowledge, the first report of its antitumor activity in gastric cancer.

Rather than isolating specific compounds, this study focused on evaluating the biological activity of crude extracts, aligning with a phytotherapeutic approach that considers the synergistic interactions among multiple natural constituents. Crude extracts are widely used in traditional medicine and modern herbal-based formulations, and their activity may not be attributable to a single compound but to the complex chemical interplay within the matrix. This strategy allows the identification of promising plant-based extracts for further development, whether as standardized phytoproducts or as the basis for compound isolation and drug design.

Therefore, this work focused on demonstrating *in vitro* the biological activity on stomach cancer cells of derivatives obtained from two promising plants, *P. volubilis*, and *M. oleifera*, which have been attributed several benefits for human health, exploring different biological approaches to demonstrate their antitumor capacity.

The viability and proliferation of AGC tumor cells were affected by all biological derivatives, and inhibition of proliferation was maintained over time. *M. oleifera* and *P. volubilis* derivatives have cytotoxic activity for AGS tumor cells and can inhibit the proliferation of these cells during the first 96 hours after treatment. This effect may be due to a wide range of factors related to the preservation of phytochemicals, the percentage of water in the leaves, and the dissolution process to which the biological material is subjected. For example, solvents with more apolar and hydrophobic structures have a more significant effect at lower concentrations because of their ability to potentiate anticancer metabolites such as kaempferol, niazimycin, β-sitosterol-3-O-β-D-glucopyranoside and benzyl isothiocyanate present in *M. oleifera* leaf ( Padayachee & Baijnath, 2020). Solvent polarity plays a critical role in the solubility of compounds, especially phenolics, as less polar solutes solubilize better in less polar solvents and more polar solutes in more polar solvents ( Chahardehi, Arsad, Ismail, & Lim, 2021). Moreover, many biomolecules with cytotoxic and antiproliferative activity are inefficiently obtained with polar or hydrophilic solvents due to the presence of phenolic metabolites or hydrophobic residues. For this reason, organic solvents are often preferred in proliferation approaches ( Lezoul, Belkadi, Habibi, & Guillén, 2020). However, there is evidence of significant anti-inflammatory and antioxidant activity of ethanolic extracts obtained from plants compared to organic solvents ( Truong et al., 2019), as well as high *in vitro* antiproliferative capacity towards breast cancer cells (M. Khalil et al., 2021) and colorectal cancer lines ( Mesas et al., 2021). In the specific case of *Moringa*, the ethanolic extract obtained from its seeds exhibited greater antiproliferative power than other extracts toward colon tumor lines, as demonstrated by Fuel et al. in 2021 ( Fuel et al., 2021). These findings and those obtained in our study reinforce the fact that ethanolic extracts from different parts of plants promote biological effects of interest due to the different concentrations of flavonoids found in them ( Xu, Chen, & Guo, 2019).

In the case of *P. volubilis* oil, which did not require chemical processing to obtain it, absolute cell culture death was observed at the highest dose tested. This event did not occur with the extracts in the viability assays. There is little evidence on the antitumor role of *P. volubilis* derivatives. An anti-hepatoma effect has been reported with peptides derived from the plant’s seed ( He et al., 2023) and inhibition of cell growth in A459 and Hela lines due to methanolic and hexane extracts of its leaves, respectively, almost halving the cell population at the end of the assays ( Nascimento et al., 2013). At the time of writing, there is no information on the *in vitro* antitumor potential of *P. volubilis* oil seeds.

The seed of *P. volubilis* has a particular chemical composition with a high amount of polyunsaturated fatty acids (PUFAs), the most important of which are linolenic acid (LA) and α-linolenic acid (ALA), which make up around 80% of these PUFAs ( Cárdenas et al., 2021). Other vital metabolites in the oil include tocopherols, phytosterols, and terpenoids. The latter is responsible for vegetable oils’ physical characteristics; some have demonstrated antitumor effects, such as aristolene ( da Anunciaçao et al., 2020) and cycloartenol ( Niu et al., 2018). On the other hand, phytosterols and anticancer evidence exist for β-sitosterol ( Bin Sayeed & Ameen, 2015), phytol ( Alencar et al., 2018), and stigmasterol, which are some of the most abundant sterols in plant oil ( Ramos-Escudero et al., 2019).

Li et al. identified the anticancer effects of stigmasterol on cell viability and proliferation in gastric cancer cells through mechanisms associated with the disruption of apoptosis ( Li et al., 2018). Our findings show similarities with these results. Nonetheless, in Li’s study, they observed a G2/M phase arrest ( Li et al., 2018). We observed that *P. volubilis* oil is a cell death-inducing agent favoring an increase of cells in the sub-G1 phase in a statistically significant way. In addition, all derivatives of *M. oleifera* and *P. volubilis* leaves, but also *P. volubilis* oil, induced caspase activity in AGS tumor cells, suggesting that apoptosis is the cytotoxic mechanism of cell death *.* The sub-G1 DNA content is characteristic of apoptotic cells, which further reinforces the cytotoxic and antiproliferative effect of the oil on the cells tested.

Studies of apoptotic markers such as phosphatidylserine externalization and caspase activity were performed to decipher the mechanisms responsible for cell death. In the early stages of cell death, translocation of phosphatidylserine to the outer layer of the cell membrane is observed, an event identified in our study by forming a luminescent complex with annexin V through luciferase conjugation. At later stages, destabilization of the cell membrane occurs, allowing entry of a fluorescent probe that binds to nucleic acids, indicating post-apoptotic necrosis, an apoptotic phenotype that results from a kinetic mismatch between an initial luminescent signal and a subsequent fluorescent signal.

According to the results obtained, the assays that faithfully represented the above described were the treatment of tumor cells with MOPE and PVEtOH extracts. For the rest of the extracts and the oil, an atypical behavior was observed in the curves, in which the first signal detected was fluorescence followed by luminescence. When comparing the signals of all derivatives with respect to the controls, especially the negative, it is inferred that in the former, other plausible mechanisms of cell death are occurring in these cells despite not exhibiting the orthodox apoptotic phenotype of this approach.

A possible cause of this atypia could be related to the time used for the analysis of the signal emission. Several assays of this type are based on the kinetic analysis of the signals in a time span of no more than 24, with short time ranges between detections (30 mins). It is very likely that these short kinetics provide a “zoom in” to events occurring in the earliest phase of treatment, thus increasing the likelihood of identifying the expected apoptotic phenotype. In any case, it should be taken into account that in some cases, as in ROS-mediated cell damage, some phytoelements can lead to the concurrence of two death mechanisms (apoptosis and necrosis) simultaneously. Among these phytoelements, vanicosides have been found, among other polyphenols, which can provoke oxidative stress in tumor cells and thus lead to cell death through various mechanisms ( Bian, Wei, Zhao, & Li, 2020).

Regarding the caspase study, a higher activity of caspase 8 was observed in comparison with the rest of the enzymes, a sign indicative of the involvement of the extrinsic pathway of apoptosis, especially in the cells treated with the oil, since in these cells the enzyme was activated to a greater extent than in the other groups. Similarly, discrete involvement of the intrinsic pathway was identified through the activation of caspase 9 and the activation of caspase 3 confirmed apoptotic death in all groups, especially in the group treated with PVEtOH.

The cascades involved in the two pathways are different but converge in the activation of caspases. Initiation of the extrinsic pathway requires a trigger, which could be a phytochemical acting as a ligand, which subsequently binds to the death receptor (FAS) on the cell surface to form a complex involving intracellular activation of initiator caspase 8 with subsequent activation of executioner caspase 3 ( Lavrik, Golks, & Krammer, 2005). According to the findings of this study, both extracts and oil function as inducers of cell death through the extrinsic pathway.

Several elements or signals can exert an activating role on proapoptotic events, among them ROS and NO. We decided to explore the induction of this type of molecules in tumor cells around *Plukenetia Volubilis* oil, since it was this derivative that had been exhibiting interesting characteristics in terms of toxicity, antiproliferation, caspases activation and cell cycle arrest, adding to this decision the fact that there are no reported antecedents on the induction role of metabolic stress in cancer for this derivative.

Treatment with *P. volubilis* oil leads to oxidative stress in AGS cells. The data obtained showed an evident increase in ROS and NO production in the treated tumor cells with the oil compared to the untreated group to a relatively similar degree to that induced by the positive control, especially for reactive nitrogen species. The role of NO as a tumor inductor molecule is ambiguous in comparison with ROS, as its effects depend on its concentrations and origin. At low concentrations it may exert a cytoprotective effect but at high levels it has been shown to act as a propapoptotic agent ( Korde Choudhari, Chaudhary, Bagde, Gadbail, & Joshi, 2013). All these nitrosative alterations in DNA force cells to activate their repair mechanisms either by entering senescence pathways, or if the damage is very deep, apoptosis ( Nakanishi, Shimada, & Niida, 2006), as observed in AGS cells according to the results obtained in cytotoxicity analysis, proliferation and apoptosis studies developed in our work.

In order to further explore the other capabilities of these plant derivatives, we sought to explore their ability to block the migration of AGS cells by studying changes in their motility. Of all the products studied, only MOEtOH had a notable effect in inhibiting the motility of these cells. According to the data obtained for each group in relation to the distance between the edges of the wounds between 0 and 48 hours, it could be established that the recovery of the wound in the untreated cells was 66% while in those treated with the extract it was 48%. Although without statistical significance, these findings indicate that at least this extract contributes to the change of phenotype in stomach tumor cells with the consequent affectation in the migration capacity. Cell motility is a hallmark of tumor progression and loss of this property has been associated with improved prognosis. However, further studies are needed to establish whether the cells are incited by the action of the extracts to follow an apoptotic or antimetastatic pathway, such as a reversal of the epithelial-mesenchymal transition. However, further studies are needed to establish whether the cells are incited by the action of the extracts to follow an apoptotic or antimetastatic pathway, such as a reversal of the epithelial-mesenchymal transition. In an approach similar to that performed in our study, Shu X et al. (2018) were able to demonstrate at the cellular and molecular level the hypothesis related to the reversal of epithelial-mesenchymal transition in gastric cancer cells. In that work, extracts from *M. oleifera* seed were evaluated, and through migration and invasion assays the anti-metastatic potential in gastric cancer cells was evidenced, also revealing the possible cellular target of the extract, since a positive regulation of NDRG1 expression was observed in the treated cells ( Shu et al., 2018), which is a gene with invasion and metastasis suppressor activity. Based on this evidence, gene expression assays or protein analysis could be performed in the cells treated with our extract, contemplating not only the evaluation of NDRG1 but also other reversion markers such as ZO-1 and vimentin, among others.

**Figure 9.**
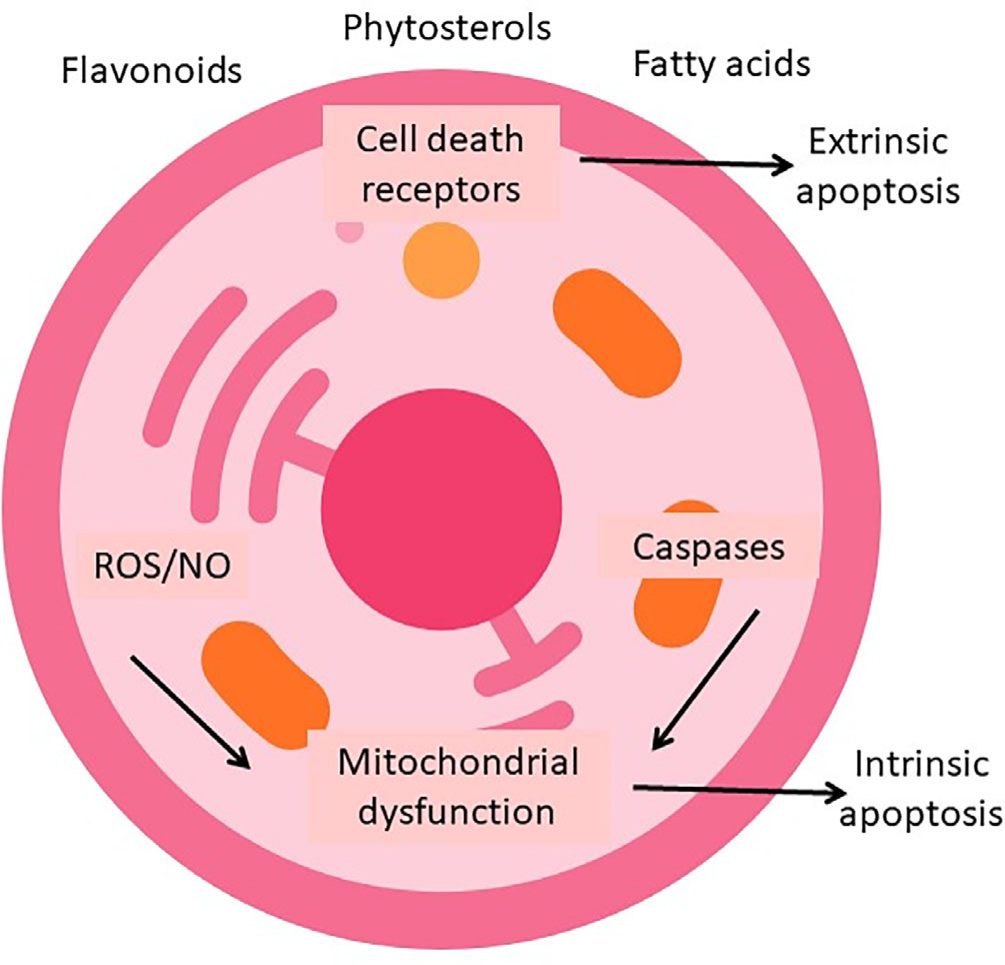
Proposed mechanism of synergistic antitumor activity of crude plant extracts. The diagram illustrates how key phytochemicals present in *Moringa oleifera* and *Plukenetia volubilis* extracts, such as flavonoids, polyunsaturated fatty acids, and phytosterols, may act on complementary cellular targets. These include induction of oxidative and nitrosative stress (ROS/NO), activation of death receptors (extrinsic apoptosis), mitochondrial dysfunction (intrinsic apoptosis), and caspase cascade activation. The convergence of these mechanisms enhances cytotoxicity in gastric cancer cells, supporting the hypothesis of synergistic activity in crude extracts.

The observed biological activity may result from the synergistic interplay of various phytochemicals naturally co-occurring in the extracts, such as flavonoids, polyunsaturated fatty acids, and phytosterols. Synergy can arise when compounds act on multiple molecular targets within the same pathway or on different but converging pathways. Flavonoids may induce mitochondria-dependent apoptosis, and sterols can modulate membrane integrity as well as the sensitivity of death receptors. Fatty acids, on the other hand, can increase ROS-mediated stress. Together, these interactions can amplify cytotoxic signals and circumvent resistance mechanisms. Although not directly tested in this study, such multi-target synergy has been proposed as a key feature of crude plant extracts in cancer therapeutics. A proposed model of these interactions is presented in Figure 9.

To our knowledge, this is the first report demonstrating the in vitro antitumor activity *of P. volubilis* seed oil against gastric cancer cells, including its effects on proliferation, apoptosis induction, cell cycle arrest, and oxidative stress. While previous studies have explored the bioactivity of *P. volubilis* seed peptides and leaf extracts in other cancer models, such as liver, lung, or cervical carcinoma, no studies have addressed the direct cytotoxic and pro-apoptotic effects of the unprocessed seed oil in gastric cancer. In addition, this study is also one of the few that comparatively evaluates ethanolic and oil extracts from two different plants ( *P. volubilis* and *M. oleifera*) in a unified experimental model, offering a broader understanding of their mechanisms of action. Our findings provide new evidence supporting the role of these natural derivatives as potential therapeutic agents for gastric cancer and offer a starting point for further investigation into their molecular targets and *in vivo* efficacy.

Overall, this study makes original contributions to the field of natural product research in oncology. This work can be considered as one of the first to report the antitumor activity of *Plukenetia volubilis* seed oil in gastric cancer cells, and one of the few that directly compares ethanolic and oil-based extracts from two botanically unrelated species in a unified experimental model. Identifying extrinsic apoptosis activation, induction of oxidative stress, and cell cycle arrest highlights key mechanistic insights. These findings support the continued investigation of these derivatives as phytotherapeutic agents and as lead candidates for developing novel, low-toxicity treatments against gastric cancer.

Nonetheless, using crude extracts limits the ability to attribute the observed biological effects to specific active compounds, and the potential contribution of individual constituents was not experimentally determined. Second, all experiments were conducted in a single gastric cancer cell line (AGS), and therefore, the findings may not fully represent other tumor types or gastric cancer subtypes. Although evidence of apoptosis and oxidative stress was identified, more in-depth molecular validation (e.g., western blotting, gene expression profiling) was not performed. Lastly, *in vivo* studies are needed to confirm the extracts safety, efficacy, and pharmacokinetics under physiological conditions. Despite these limitations, the present findings provide a valuable foundation for further research on the anticancer potential of *M. oleifera* and *P. volubilis*.

## Ethics and consent

Ethical approval and consent were not required.

## Data Availability

Figshare: Data paper.xlsx, https://doi.org/10.6084/m9.figshare.27317220.v1 ( Vargas, 2024). This project contains the following underlying data:
• Data paper.xlsx Data are available under the terms of the Creative Commons Attribution 4.0 International license (CC-BY 4.0).
