# Supplementary material for: In vitro antitumor capacity of extracts obtained from the plants Plukenetia volubilis (Sacha inchi) and Moringa oleifera in gastric cancer
Source: F1000Res. 2025 May 27;14:194. Originally published 2025 Feb 12. [Version 2] doi: 10.12688/f1000research.158563.2 (PMC12120421; doi:10.12688/f1000research.158563.2)
Supplement: Peer review history

# Reviewer report: Reviewer response for version 2

**DOI:** 10.5256/f1000research.182185.r387435

**Published:** 2025-06-13

**Authors:** Rabia Yılmaz Öztürk

**Affiliations:** 1 Yildiz Technical University, Istanbul, Turkey

**Below, I have listed the deficiencies that the author claimed to have corrected in response to the referee’s comments, but which I could not verify. I kindly request that you either provide the corrected version or implement the necessary corrections if they have not yet been made.**

**Intro**

 As I mentioned before, the sentence below is not relevant to the article. In my opinion, it needs to be removed from the introduction section. If the sentence remains, you must explain why you wrote it.

 "Between 1971 and 2007, an increase in survival of only 17% has been achieved in ovarian cancer (Lloyd, Cree, & Savage, 2015), while in breast cancer, the increase has been 38% in 10-year survival."

**Methods**

 1-Spelling errors must be corrected (for example, H2DCFDA). It should be fixed like this: H _2_DCFDA **.**

 2- "ON production" Abbreviations should be written clearly.

**Results**

 1- The acronym for NO should be given

 2- The explanation of Table 2 is insufficient. What is meant by C- or C+?

 4- I could not identify where the extract is mentioned in the results of the wound healing test.

 In addition, which extract used should be written in the method or results section.

 5- All figure descriptions must specify the extracts and their respective concentrations used in the experiment.

 6- In all analyses, all extracts used in the study must be included, or the reason why they were not used must be explained in detail. In addition, which extract used should be written in the method or results section. For each analysis, results of both extracts should be given, even if there is no effect. 

Is the work clearly and accurately presented and does it cite the current literature?

Partly

If applicable, is the statistical analysis and its interpretation appropriate?

Yes

Are all the source data underlying the results available to ensure full reproducibility?

Partly

Is the study design appropriate and is the work technically sound?

Yes

Are the conclusions drawn adequately supported by the results?

Yes

Are sufficient details of methods and analysis provided to allow replication by others?

Yes

Reviewer Expertise:

Anticancer agent, cancer

I confirm that I have read this submission and believe that I have an appropriate level of expertise to confirm that it is of an acceptable scientific standard, however I have significant reservations, as outlined above.

# Reviewer report: Reviewer response for version 2

**DOI:** 10.5256/f1000research.182185.r387434

**Published:** 2025-06-03

**Authors:** Hassan A. Alshamsi

**Affiliations:** 1 University of Al-Qadisiyah, Al Diwaniyah, Iraq

I found that the authors addressed all the comments and improved the manuscript. The updated version is acceptable for indexing in F1000Research.

Is the work clearly and accurately presented and does it cite the current literature?

Yes

If applicable, is the statistical analysis and its interpretation appropriate?

Yes

Are all the source data underlying the results available to ensure full reproducibility?

Partly

Is the study design appropriate and is the work technically sound?

Yes

Are the conclusions drawn adequately supported by the results?

Yes

Are sufficient details of methods and analysis provided to allow replication by others?

Yes

Reviewer Expertise:

Green synthesis, antibacterial activity, anticancer activity, photochemistry

I confirm that I have read this submission and believe that I have an appropriate level of expertise to confirm that it is of an acceptable scientific standard.

# Reviewer report: Reviewer response for version 2

**DOI:** 10.5256/f1000research.182185.r387433

**Published:** 2025-05-28

**Authors:** Abdul Mueed

**Affiliations:** 1 Nanjing Agricultural University, Nanjing, China; 2 State Key Laboratory of Food Science and Nutrition, Nanchang University, Nanchang, Jiangxi, China

I agree with author response [new version].

Is the work clearly and accurately presented and does it cite the current literature?

No

If applicable, is the statistical analysis and its interpretation appropriate?

Yes

Are all the source data underlying the results available to ensure full reproducibility?

Yes

Is the study design appropriate and is the work technically sound?

Yes

Are the conclusions drawn adequately supported by the results?

Yes

Are sufficient details of methods and analysis provided to allow replication by others?

Yes

Reviewer Expertise:

lipid oxidation, metabolism and anticancer activity

I confirm that I have read this submission and believe that I have an appropriate level of expertise to confirm that it is of an acceptable scientific standard.

# Reviewer report: Reviewer response for version 1

**DOI:** 10.5256/f1000research.174174.r375094

**Published:** 2025-05-06

**Authors:** Abdul Mueed

**Affiliations:** 1 Nanjing Agricultural University, Nanjing, China; 2 State Key Laboratory of Food Science and Nutrition, Nanchang University, Nanchang, Jiangxi, China

Dear author

 The research “In vitro antitumor capacity of extracts obtained from the plants *Plukenetia volubilis *(Sacha inchi) and *Moringa oleifera* in gastric cancer” is interesting and comprehensively reports the anticancer activity of *Plukenetia volubilis* (Sacha inchi) and *Moringa oleifera* extracts. The manuscript is well-structure but some suggestions and comments are listed for the author to improve the quality of the manuscript.

 Introduction section: the author should need to clearly state the research gap regarding the *Plukenetia volubilis* (Sacha inchi) and *Moringa oleifera* extracts, and anticancer activity. And emphasized the novelty of the research.

 Clearly articulate the hypothesis that *Plukenetia volubilis* (Sacha inchi) and *Moringa oleifera* extract regulate anticancer activity leading to proliferation, migration and cell cycle arrest etc.

 Use the following two articles to articulate the introduction with the hypothesis and objective of the study

 Mueed A, et al., 2023 (Ref 1)  

 Shahid A, et al., 2022 (Ref 2)

 Figure 8. Inhibition of migration of AGS cells. The microphotographs magnification is not the same, please provide the same magnification mentioned in the figure legend.

 Results section, Inhibition of cell migration is not well explained

 Why just use the ethanolic extract at a concentration of 100 μg/ml, while *M. oleifera* cyclohexane extract, which showed a CC _50_ of 94.3 μg/mL, why not use the cyclohexane extract?

 Figure 4. Effect of extracts and oil on the viability/cytotoxicity of AGS cells. The microphotograph magnification is not the same, please provide the same magnification mentioned in the figure legend.

Is the work clearly and accurately presented and does it cite the current literature?

No

If applicable, is the statistical analysis and its interpretation appropriate?

Yes

Are all the source data underlying the results available to ensure full reproducibility?

Yes

Is the study design appropriate and is the work technically sound?

Yes

Are the conclusions drawn adequately supported by the results?

Yes

Are sufficient details of methods and analysis provided to allow replication by others?

Yes

Reviewer Expertise:

lipid oxidation, metabolism and anticancer activity

I confirm that I have read this submission and believe that I have an appropriate level of expertise to confirm that it is of an acceptable scientific standard, however I have significant reservations, as outlined above.

## References

[1] : Extraction, characterization of polyphenols from certain medicinal plants and evaluation of their antioxidant, antitumor, antidiabetic, antimicrobial properties, and potential use in human nutrition. *Front Nutr* .2023;10: 10.3389/fnut.2023.1125106 1125106 10.3389/fnut.2023.1125106 37415912 PMC10320526

[2] : Phytochemical Profiling of the Ethanolic Extract of Zaleya pentandra L. Jaffery and Its Biological Activities by In-Vitro Assays and In-Silico Molecular Docking. *Applied Sciences* .2022;13(1) : 10.3390/app13010584 10.3390/app13010584

# Reviewer report: Reviewer response for version 1

**DOI:** 10.5256/f1000research.174174.r375096

**Published:** 2025-04-15

**Authors:** Hassan A. Alshamsi

**Affiliations:** 1 University of Al-Qadisiyah, Al Diwaniyah, Iraq

The manuscript entitled "In vitro antitumor capacity of extracts obtained from the plants *Plukenetia volubilis *(Sacha inchi) and *Moringa oleifera *in gastric cancer" was reviewed. It details the experimental extraction of anticancer compounds from plants native to Columbia. This manuscript describes 3 plant extracts against gastric cancer. Overall, the manuscript is interesting, well written and well organized and the methodology is appropriate. This is good preliminary work which leads onto other in vitro  studies and the data would be of interest to the readers of the journal. However, some essential revisions must be performed based on the following comments. In my opinion, this manuscript can be accepted in this journal after making the required modifications.

**Comments**

**Introduction**

1. The novelty of the manuscript and its differences from previous related research must be emphasized.
2. Why  the authors specifically chose two medicinal plants ( *Plukenetia volubilis* (Sacha inchi) and *Moringa oleifera*) should be justified more clearly in the introduction section.
3. In the Introduction section, there is no relevant article explaining the composition of phytochemicals in plant extracts. Are there many studies that have addressed this topic?
4. Since the work focuses on the preparation of plant extracts, are these extracts colored? This may significantly impede the performance of the spectrophotometric measurement, so it is important to know this.

**Methods**

1. If authors can determine which component of the extract has the most potent anticancer effect, the research will be more useful to readers. In addition, testing the crude extract alone may yield inaccurate results due to the potential synergistic or antagonistic effects of individual components, leading to inaccurate conclusion. To accurately evaluate natural anti-cancer compounds, it is recommended to fractionate the crude extract and perform chemical analysis using techniques such as LC/MS to obtain a more accurate identification.
2. The extraction processes are not entirely clear, the concentration of the ether, cyclohexane and ethanol extracts must mentioned in details such as volume of solvent and time of extraction.
3. All methods should be referenced.

**Results**

1. Please unify the volume unit as mL.
2. The magnification power of Figures 1 and 2 should be mentioned.
3. The significance and progress of the results of this study should be stated more clearly in the Results section.
4. The standard deviation values (Table 2) ​​with treatment C+ appear to be big compared to the treatment C-.

**Discussion**

1. As for the first two paragraphs in the discussion section, they are not appropriate and are formulated in the style of an introduction.
2. How can the synergistic activity of the phytochemicals be explained? The author should state the molecular mechanism behind this activity. Draw a suitable diagram for this purpose
3. It is suggested that authors indicate the limitations of the study at the end of the discussion

Is the work clearly and accurately presented and does it cite the current literature?

Yes

If applicable, is the statistical analysis and its interpretation appropriate?

Yes

Are all the source data underlying the results available to ensure full reproducibility?

Partly

Is the study design appropriate and is the work technically sound?

Yes

Are the conclusions drawn adequately supported by the results?

Yes

Are sufficient details of methods and analysis provided to allow replication by others?

Yes

Reviewer Expertise:

Green synthesis, antibacterial activity, anticancer activity, photochemistry

I confirm that I have read this submission and believe that I have an appropriate level of expertise to confirm that it is of an acceptable scientific standard, however I have significant reservations, as outlined above.

# Reviewer report: Reviewer response for version 1

**DOI:** 10.5256/f1000research.174174.r366675

**Published:** 2025-03-03

**Authors:** Rabia Yılmaz Öztürk

**Affiliations:** 1 Yildiz Technical University, Istanbul, Turkey

**Introduction**

 The sentence below is not relevant to the article. 

 "Between 1971 and 2007, an increase in survival of only 17% has been achieved in ovarian cancer ( Lloyd, Cree, & Savage, 2015), while in breast cancer, the increase has been 38% in 10-year survival."

**Methods**

 1- The spelling of roto evaporation should be checked.

 2- In the method section, using dots and commas in numbers should be checked.

 3- Space usage should be corrected (for example, "100μl in each well. 20μl/....".).

 4- Why were different concentrations used for cell cytotoxicity and cell proliferation?

 5- Spelling errors must be corrected (for example, H2DCFDA).

 6- What is the ON production?

**Results**

 1- The acronym for NO should be given

 2- The explanation of Table 2 is insufficient. What is meant by C- or C+?

 3- Why was only *Plukenetia Volubilis* seed oil examined in the ROS experiment, and why were other extracts not analyzed?

 4- Which extract was used in the wound closure test (Figure 8)? Why are there no other extract results analyzed in the study?

 5- All figure descriptions must specify the extracts and their respective concentrations used in the experiment.

 6- In all analyses, all extracts used in the study must be included, or the reason why they were not used must be explained in detail.

**Discussion**

 1- An article (Shu X, et al., 2018 [Ref 1]) examining the effects of *Moringa oleifera* extracts on gastric cancer and its metastasis should be included in the discussion section and discussed.

Is the work clearly and accurately presented and does it cite the current literature?

Partly

If applicable, is the statistical analysis and its interpretation appropriate?

Yes

Are all the source data underlying the results available to ensure full reproducibility?

Partly

Is the study design appropriate and is the work technically sound?

Yes

Are the conclusions drawn adequately supported by the results?

Yes

Are sufficient details of methods and analysis provided to allow replication by others?

Yes

Reviewer Expertise:

Anticancer agent, cancer

I confirm that I have read this submission and believe that I have an appropriate level of expertise to confirm that it is of an acceptable scientific standard, however I have significant reservations, as outlined above.

## References

[1] : Extract from Moringa oleifera seeds suppresses the epithelial-mesenchymal transition-mediated metastasis of gastric cancer by targeting the metastatic suppressor NDRG1. *Journal of Functional Foods* .2018;50: 10.1016/j.jff.2018.09.030 93-103 10.1016/j.jff.2018.09.030

# Response

**Published:** 2025-03-11

**Authors:** Javier Soto

**Affiliations:** Norte de Santander, Universidad de Santander, Cucuta, Norte de Santander, Colombia

Dear reviewer

 We sincerely appreciate the time and effort that you and the reviewers have dedicated to evaluating our manuscript, *In vitro antitumor capacity of extracts obtained from the plants Plukenetia volubilis (Sacha inchi) and Moringa oleifera in gastric cancer*. We are grateful for the useful comments and constructive feedback, which have helped us to improve the clarity, rigor, and impact of our work.

 We have carefully considered all the suggestions and have made the necessary revisions accordingly. Below, we provide a detailed response to each comment, highlighting the changes made to the manuscript. For clarity, we present the reviewers' comments in bold, followed by our responses in regular text. In cases where substantial modifications were made, we indicate the corresponding changes in the revised manuscript.

**Introduction**

**The sentence below is not relevant to the article.**

**"Between 1971 and 2007, an increase in survival of only 17% has been achieved in ovarian cancer (** **Lloyd, Cree, & Savage, 2015** **), while in breast cancer, the increase has been 38% in 10-year survival."**

 Response: We agree with this observation. Text was removed from the document.

**Methods**

**1- The spelling of roto evaporation should be checked.**

*Response: Corrected*

**2- In the method section, using dots and commas in numbers should be checked.**

*Response: Revised and corrected*

**3- Space usage should be corrected (for example, "100** **μ** **l in each well. 20** **μ** **l/....".).**

*Response: Revised and corrected.*

**4- Why were different concentrations used for cell cytotoxicity and cell proliferation?**

*Response: We consider it inadequate to evaluate the same concentrations since cytotoxicity and cell proliferation are distinct events. Cytotoxicity assays assesses cell damage or death, whereas proliferation measures cell growth and replication. A concentration that induces cytotoxicity may not be the same as one that affects proliferation.*

*When we adapt the concentration range based on the effect being studied, we can obtain more precise and relevant information while also enabling the establishment of a dose-response curve. This helps to identify the minimum effective concentration and the maximum tolerable dose, optimizing the interpretation of the extract’s effects. In some cases, some concentrations may stimulate cell proliferation instead of inducing toxicity, so evaluating different ranges allows for determining optimal conditions to maximize the desired effect.*

*Some concentrations may inhibit proliferation without causing cytotoxicity, whereas others may stimulate cell growth without significant damage. Therefore, depending on the study's objectives, it is recommended to use a broader range for cytotoxicity assessment and a more specific range for cell proliferation to obtain clearer results.*

*Adjusting the concentration range according to the type of evaluation (cytotoxicity or proliferation) enables the collection of more precise and reproducible data, improving result interpretation and its applicability in future research.*

*In our work, initially a range of concentrations was used to identify the CC50 in the cytotoxicity tests, but later in the proliferation assays the concentrations were in ranges lower than the CC50 because we wanted to evaluate if lower concentrations could have an antiproliferative effect when the cells are treated for a longer time, as it happens in the proliferation assays compared to the cytotoxicity assays. In fact, proliferation assays are usually performed with a single dose, but we wanted to demonstrate the effect already described*.

**5- Spelling errors must be corrected (for example, H2DCFDA).**

*Response: revised and corrected*

**6- What is the ON production?**

*Response: ON means nitric oxide. There was a spelling mistake. Now was revised and corrected.*

**Results**

**1- The acronym for NO should be given**

*Response: Acronym for NO was given in text.*

**2- The explanation of Table 2 is insufficient. What is meant by C- or C+?**

*Response: The table and explanation have been modified*

 Treatment    ROS    ON

 Negative control     29.3 ± 2,6    22.3 ± 1.7

 Positive control     63.1 ± 13.6 (↑ 2.1 x)    58.3 ± 6.5 (↑ 2.6 x)

 P. volubilis oil    46.7 ± 10.8 (↑ 1.6 x)     50.1 ± 2.7 (↑ 2.2 x)

 Data show the mean ± SD of the percentage of cells positive for ROS and NO without treatment (Negative control), treated with PHA to induce ROS production, or PMA to induce NO production (Positive control), and treated with *P. volubilis* seed oil. Arrows ↑↓ represent the increase or decrease concerning the untreated basal condition.

**3- Why was only *Plukenetia Volubilis* seed oil examined in the ROS experiment, and why were other extracts not analyzed?**

*Response: This approach specifically focused on Plukenetia volubilis oil, as this derivative was exhibiting promising characteristics at that stage of the research, particularly in terms of toxicity, antiproliferative effects, caspase activation, and cell cycle arrest. Additionally, the decision to explore this oil was reinforced by the lack of prior studies on its role in inducing metabolic stress in cancer.*

**4- Which extract was used in the wound closure test (Figure 8)? Why are there no other extract results analyzed in the study?**

*Response: As indicated in the results, the ethanol extract of Moringa oleifera was used in the wound closure test (Figure 8). This was the only extract analyzed in the study because it was the only one that exhibited notable antimigratory activity in the treated cells.*

**5- All figure descriptions must specify the extracts and their respective concentrations used in the experiment.**

*Response: The required information was added.*

**6- In all analyses, all extracts used in the study must be included, or the reason why they were not used must be explained in detail.**

*Response: This concern were addressed in questions 3 and 4. Please see above.*

**Discussion**

**1- An article (Shu X, et al., 2018 [Ref 1]) examining the effects of *Moringa oleifera* extracts on gastric cancer and its metastasis should be included in the discussion section and discussed.**

 Response: We added this paragraph:

*However, further studies are needed to establish whether the cells are incited by the action of the extracts to follow an apoptotic or antimetastatic pathway, such as a reversal of the epithelial-mesenchymal transition in gastric cancer cells. In an approach similar to that performed in our study, Shu X et al (2018) were able to demonstrate at the cellular and molecular level the hypothesis related to the reversal of epithelial-mesenchymal transition. In that work, extracts from M. oleifera seed were evaluated, and through migration and invasion assays the anti-metastatic potential in gastric cancer cells was evidenced, also revealing the possible cellular target of the extract, since a positive regulation of NDRG1 expression was observed in the treated cells* *(Shu et al., 2018)* *, which is a gene with invasion and metastasis suppressor activity. Based on this evidence, gene expression assays or protein analysis could be performed in the cells treated with our extract, contemplating not only the evaluation of NDRG1 but also other reversion markers such as ZO-1 and vimentin, among others.*
